# Supplementary material for: The effect of rehabilitation time on functional recovery after arthroscopic rotator cuff repair: a systematic review and meta-analysis
Source: PeerJ. 2024 May 20;12:e17395. doi: 10.7717/peerj.17395 (PMC11114118; doi:10.7717/peerj.17395)
Supplement: Supplemental Information 2 [file peerj-12-17395-s002.docx]

**Supplementary Table 1 Scores assessing the methodological quality**

| Eligibility criteria  specified | Arndt J  2012 | Cuff  2012 | Kim  2012 | Lee  2012 | Keener  2014 | Sheps  2015 | Mazzocca  2017 | Huang  2015 | Guo  2019 |
| --- | --- | --- | --- | --- | --- | --- | --- | --- | --- |
| Random allocation | Y | Y | Y | Y | Y | Y | Y | Y | Y |
| Concealed  Allocation | Y | Y | Y | Y | Y | Y | Y | Y | Y |
| Groups similar at  Baseline | Y | Y | Y | Y | Y | N | Y | Y | Y |
| Subject blinding | N | N | N | N | N | Y | Y | N | N |
| Therapist blinding | N | N | N | N | N | Y | Y | N | N |
| Assessor blinding | Y | Y | Y | Y | Y | Y | Y | Y | Y |
| Less than %15  Dropouts | Y | Y | Y | Y | Y | Y | N | Y | Y |
| Intention to treat  Analysis | Y | N | Y | N | Y | Y | Y | N | N |
| Between-group  statistical  comparisons | Y | Y | Y | Y | Y | Y | Y | Y | Y |
| Point measures and  variability data | N | Y | Y | Y | N | Y | Y | Y | Y |
| Total PEDro score | 7 | 7 | 8 | 7 | 7 | 9 | 9 | 7 | 7 |
