# Supplementary material for: The effect of rehabilitation time on functional recovery after arthroscopic rotator cuff repair: a systematic review and meta-analysis
Source: PeerJ. 2024 May 20;12:e17395. doi: 10.7717/peerj.17395 (PMC11114118; doi:10.7717/peerj.17395)
Supplement: Supplemental Information 4 [file peerj-12-17395-s004.docx]

1. The rationale for conducting the systematic review / meta-analysis

**Respond：**The best time to recuperate after arthroscopic treatment is still being discussed. Early mobilization proponents stress that this significantly lowers postoperative shoulder stiffness, a frequent consequence after RCR. On the other hand, other studies disagree, contending that ankylosis may not increase with longer braking durations. Delaying exercise improves rotator cuff shape, composition, and biomechanical qualities while lowering stress at the healing site and lowering the chance of recurrent soft tissue re-dissection, according to animal studies . The goals of optimal rehabilitation programs are to restore normal shoulder function, allow tendons to recover, and prevent reinjury . Options for early and delayed recovery have been made to accomplish this aim. After rotator cuff repair, immobilization is advised for 6 to 8 weeks, according to the deferred rehabilitation treatment plan. The information from animal research demonstrating that the tendon healing process takes 4 to 16 weeks is the basis for the theoretical rationale behind this strategy. Although early rehabilitation programs that permit activity to start on the first postoperative day theoretically minimize postoperative stiffness and muscle atrophy, some research indicates that in 20% to 90% of instances, they increase the chance of tendon re-injury. Clinical trials with varying designs yield inconsistent findings on the best rehabilitation duration and cannot generate substantial proof about the best rehabilitation regimen following RCR.In this study, we compared the effects of early and delayed rehabilitation on the function of patients after rotator cuff repair by Meta-analysis to find effective interventions to promote the recovery of shoulder function.

1. The contribution that it makes to knowledge in light of previously published related reports, including other meta-analyses and systematic reviews.

**Respond：**According to the conclusion of this meta-analysis, we believe that the improvement in shoulder function following arthroscopic rotator cuff surgery does not differ clinically between early and delayed rehabilitation. When implementing rehabilitation following rotator cuff repair, it is essential to consider the paradoxes surrounding shoulder range of motion and tendon anatomic healing. A program that allows for flexible progression based on the patient's ability to meet predetermined clinical goals or criteria may be a better option.
